# Aerial Imaging-Based Soiling Detection System for Solar Photovoltaic Panel Cleanliness Inspection

**DOI:** 10.3390/s25030738

**Published:** 2025-01-25

**Authors:** Umair Naeem, Ken Chadda, Sara Vahaji, Jawad Ahmad, Xiaodong Li, Ehsan Asadi

**Affiliations:** 1 Department of Mechanical, Manufacturing and Mechatronics Engineering, RMIT University, Melbourne, VIC 3083, Australia; umair.naeem@student.rmit.edu.au (U.N.); sara.vahaji2@rmit.edu.au (S.V.); xiaodong.li@rmit.edu.au (X.L.); 2 Yellowfin Robotic Solutions Melbourne, Melbourne, VIC 3000, Australia; ken@yellowfinrobotics.com; 3 Cybersecurity Center, Prince Mohammad Bin Fahd University, Al Khobar 31952, Saudi Arabia; jahmad@pmu.edu.sa

**Keywords:** aerial imaging, object detection, soiling detection, PV inspection

## Abstract

Unmanned Aerial Vehicles (UAVs) integrated with lightweight visual cameras hold significant promise in renewable energy asset inspection and monitoring. This study presents an AI-assisted soiling detection methodology for inspecting solar photovoltaic (PV) panels, using UAV-captured RGB images. The proposed scheme introduces an autonomous end-to-end soiling detection model for common types of soiling in solar panel installations, including bird droppings and dust. Detecting soiling, particularly bird droppings, is critical due to their pronounced negative impact on power generation, primarily through hotspot formation and their resistance to natural cleaning processes such as rain. A dataset containing aerial RGB images of PV panels with dust and bird droppings is collected as a prerequisite. This study addresses the unique challenges posed by the small size and indistinct features of bird droppings in aerial imagery in contrast to relatively large-sized dust regions. To overcome these challenges, we developed a custom model, named SDS-YOLO (Soiling Detection System YOLO), which features a Convolutional Block Attention Module (CBAM) and two dedicated detection heads optimized for dust and bird droppings. The SDS-YOLO model significantly improves detection accuracy for bird droppings while maintaining robust performance for the dust class, compared with YOLOv5, YOLOv8, and YOLOv11. With the integration of CBAM, we achieved a substantial 40.2% increase in mean Average Precision (mAP50) and a 26.6% improvement in F1 score for bird droppings. Dust detection metrics also benefited from this attention-based refinement. These results underscore the CBAM’s role in improving feature extraction and reducing false positives, particularly for challenging soiling types. Additionally, the SDS-YOLO parameter count is reduced by 24%, thus enhancing its suitability for edge computing applications.

## 1. Introduction

In pursuit of low carbon emissions, global solar PV’s power capacity is expected to surpass that of coal by 2027, becoming the largest in the world. The cumulative solar PV capacity almost triples in the IEA forecast, growing by nearly 1500 GW over the period and exceeding natural gas by 2026 and coal by 2027 [1]. The annual solar PV capacity additions will increase yearly for the next five years. Despite the initial high cost, distributed solar PV, such as rooftop solar in buildings and warehouses, is also destined for faster growth due to higher retail electricity prices. The industrial standard lifetime for most types of Solar PV is around 25 years [2]. Solar PV panels, central to harnessing solar energy, are susceptible to efficiency losses due to environmental factors, notably soiling. Early detection of soiling on PV panels is essential for ensuring optimal energy production and a prolonged service life of the panel [3]. Therefore, to maintain the high technical and economic performance of a PV system during its lifetime, regular and cost-effective Operation and maintenance (O&M) activities are required. Environmental factors profoundly influence the efficiency and performance of PV systems, among which soiling is a critical concern. Soiling refers to the accumulation of materials, such as dust, dirt, bird droppings, pollen, and vegetation, on PV panels. These contaminants reduce light transmittance and, consequently, energy generation efficiency [4]. Several imaging methods are used for soil inspection, namely infrared thermography (IR) and, most commonly, red, green, and blue (RGB) color visual inspection, which are found in practice [5]. The characterization and impact of soiling depend on several factors, including the type of installation, geographical location, and ambient environment [6,7].

Geographically, the nature and extent of soiling vary significantly. In arid zones, dust is often the main soiling agent, while humid regions may see a more significant accumulation of organic materials such as algae and fungi. Similarly, ground-mounted PV systems are more susceptible to soil deposition than rooftop installations due to proximity to dust sources [4,6]. The dust type, particle size, and chemical composition further influence the effects of soiling on PV systems, leading to varying degrees of performance degradation. Studies have shown efficiency losses exceeding 30% due to soiling in extreme conditions, which underscores the need for site-specific mitigation strategies [6]. Among the various types of soiling, dust and bird droppings have been identified as particularly detrimental [8,9]. Dust can form a uniform layer, which reduces irradiance. At the same time, bird droppings, over time, can cause more severe long-term problems, including permanent panel damage, mainly due to the hotspots created by their accumulation [4,7,8]. In addition, the deposition rate is influenced by factors such as tilt angle, surface orientation, and wind speed, which determine soil particle accumulation and adhesion dynamics [7,10]. Given the adverse effects of soiling on energy production, effective detection and mitigation mechanisms are crucial. Advanced image-based detection systems and periodic cleaning schedules have emerged as essential solutions. However, optimizing these methods requires a comprehensive understanding of soiling patterns, local environmental conditions, and cost implications [6,10]. Integrating innovative technologies, automated cleaning systems have shown promise in mitigating soiling impacts while minimizing operational costs [7]. Existing methods for soiling detection range from manual inspections to automated image-based systems. Classical preventive maintenance strategies have been proven to be suboptimal due to the need for visual inspections on site and the lack of precision and immediacy required for efficient solar panel maintenance. Consequently, to optimize O&M cost and plant efficiency simultaneously, Data Analytics and Artificial intelligence, based on predictive and early detection strategies, are being developed for remote inspection of solar PV [11].

This study collects a dataset that contains aerial images of rooftop photovoltaic installations from the suburbs of Melbourne. It identifies two primary types of soiling in photovoltaic panels: dust and bird droppings. These types of soils require different cleaning approaches; dust can be removed with low-pressure water jets, whereas bird droppings require a specialized cleaning solution for effective removal. Detecting bird droppings remains particularly challenging due to their small size, lack of distinct spatial or color features, and tendency to cover less than 2% of the surface of a panel. In contrast, dust patches are generally larger and more evenly distributed. Moreover, as detailed in Table 1, the panels are polycrystalline with a blue background, against which the bird droppings typically appear white or greyish and often lack a defined shape. The task of accurately detecting soiling is further complicated by factors such as variations in background noise, which are present in aerial images, and occlusions caused by environmental elements.

Motivation and Research Contributions: The primary challenge lies in detecting specific soiling types, such as dust and bird droppings, which are further complicated by their variability and subtle features. Bird droppings, in particular, lack distinct spatial or color characteristics yet can cause significant efficiency losses. Bird droppings are relatively small compared to other types of dirt, such as dust areas. Existing solutions often fail as they lack tailored architectures, which are optimized for multiclass soiling detection, and the specific cleaning requirements of PV installations, thus further emphasizing the need for advanced methodologies.

To address these challenges, this study introduces a comprehensive automated soiling detection pipeline, which uses advanced computer vision techniques and UAV technology. The pipeline integrates a heavily customized SDS-YOLO (Soiling Detection System YOLOv5) architecture, which is specifically designed to detect different soil patterns on top of PV panels. The proposed pipeline begins with the input of aerial RGB images captured by the UAVs of solar panels. Initially, the pipeline employs advanced image processing algorithms to detect individual solar PV panels within these images. Each identified panel is then extracted and saved as a separate image for subsequent processing. The SDS-YOLO model, which is specifically customized in this study, takes over at this stage, detecting soiling features, including dust and bird droppings, while prioritizing the recall score overall. Upon detection of soil, the model marks the affected panels, and this information is seamlessly integrated into the original aerial image. This comprehensive approach not only identifies soiled panels but also facilitates the creation of an optimal cleaning cycle tailored to the specific maintenance needs of the PV installation. The methodology and findings of this study not only demonstrate a marked improvement in soiling detection accuracy over the manual methods but also pave the way for more efficient UAV-based solar panel maintenance strategies. The main contributions of the study are summarized as follows:

- Real-World Soiling Dataset: A comprehensive dataset comprising approximately 300 aerial RGB images of PV panels was collected through multiple UAV flights conducted under various environmental conditions. The dataset is available for public access www.ia-cobotics.com/Soiling-dataset, accessed on 20 January 2025.
- End-to-end Detection Pipeline: An end-to-end pipeline is proposed for detecting dust and bird droppings in UAV-based aerial imagery. This scheme incorporates a multistage model with a panel extractor and a soiling detector based on a custom SDS-YOLO (Soiling Detection System YOLOv5) architecture, which is an enhanced version of YOLOv5 specifically designed for soiling detection on PV panels.
- SDS-YOLO architecture: A custom architecture for the YOLOv5 detection head was developed, which features only two detection heads with custom anchor sizes tailored for each class. This design optimizes the detection rate while reducing the computational complexity and inference time of the model.
- Attention Mechanism Integration: A Convolutional Block Attention Module (CBAM) was selectively integrated into the architecture to refine feature extraction by emphasizing relevant spatial and channel-wise information for each class. This addition improves the precision of the SDS-YOLO in distinguishing complex soiling patterns.

The rest of the paper is structured as follows: Section 2 provides an extensive literature review on general and aerial object detection. Section 3 details the methodology used in the proposed approach and describes the process adopted for soil detection on photovoltaic panels. Section 4 presents and discusses the results obtained from our experimental analysis, providing insight into the efficacy of our approach. Finally, Section 5 concludes the study, reflecting on its implications and suggesting avenues for future research.

## 2. Related Work

In computer vision, object detection has experienced significant advancements through the integration of deep learning methodologies. These developments have profoundly impacted diverse applications, including agriculture, automatic inspection and monitoring of renewable power plants, and surveillance. Below is a brief review of object detection in the general context and aerial applications in the applied areas.

*General object detection:* With the recent development of deep learning, current object detectors use deep neural networks and labeled datasets for detection. In general, the You Only Look Once (YOLO) series of object detectors is the most dominant in the field of object detectors. The evolution of YOLOOLO began with YOLOv1, [12], which was revolutionary for its single-stage detection framework. YOLO processes an entire image in a single pass, predicting bounding boxes and class probabilities simultaneously, leading to significant gains in speed. In 2018, [13] released YOLOv3 with further improved accuracy by using three different scales for detection and incorporating a deeper feature extractor, Darknet-53. The significant advancement in the YOLO lineup was presented in 2020, named YOLOv5 [14]. YOLOv5’s architecture, incorporating the Cross Stage Partial Network (CSPNet), optimizes computational efficiency without sacrificing accuracy. Moreover, the model features an optimized detection head with improved bounding-box regression, which improves its object-localization capabilities. Notably, YOLOv5’s automatic optimization of anchor boxes during training allows for handling datasets with small-scale features. The latest in the lineup, YOLOv8 [15], takes advantage of these developments to enhance detection precision while simultaneously decreasing computation time using an anchor-free method. Among all YOLO series, YOLOv5 is the most suitable algorithm for real-time object detection due to its promising performance and excellent computational efficiency. Several variants of YOLOv5 (n,s,m,l,x) have the same structure but a few differences in some small modules and parameters. In this paper, YOLOv5s is adopted as the baseline model for experimentation. Alongside the YOLO series, various other models were developed, each offering unique strengths. The faster R-CNN, introduced in [16], established a benchmark in precision with its innovative Region Proposal Network (RPN), which is crucial in high-accuracy applications. Mask R-CNN, an extension of Faster R-CNN by [17], excels in object detection and pixel-level instance segmentation, making it ideal for complex visual tasks. RetinaNet, developed by [18], addresses the class imbalance with its Focal Loss function, enhancing the detection of challenging objects. Lastly, EfficientDet [19] offers efficiency, optimizing the balance between accuracy and model complexity through a compound scaling method. With their distinct approaches, these models continue to shape advancements in computer vision.

*UAV RGB-imaging object detection:* Aerial object detection poses significant challenges due to variations in orientations, lighting conditions, and the high density of small objects. UAVs equipped with high-resolution cameras have become instrumental in providing real-time diagnostics for large-scale solar PV installations. Automated image-based detection systems leveraging UAV aerial imagery have emerged as a promising solution, offering continuous and cost-effective solar installation monitoring. The benefits of UAV-based inspections in addressing soiling challenges have been highlighted in [6]. This review emphasizes that soiling, particularly dust and bird droppings, is a major cause of energy loss in PV systems. Although this review underscores the utility of UAVs for large-scale monitoring, it does not provide a detailed evaluation of detection accuracy or limitations under varying environmental conditions, leaving a gap in assessing UAV-based performance robustness. In the context of specifically addressing soiling, ref. [7] provides valuable insights into the role of UAV-based imaging in monitoring soiling patterns in diverse environmental conditions, highlighting its impact on operational efficiency. However, the study lacks an in-depth discussion on the scalability of detection algorithms. In addition, the framework does not address soiling detection with significant size differences, which is crucial for targeting cleaning strategies and optimizing maintenance operations.

In their study, ref. [20] proposed a pipeline that involves locating solar panels within drone-captured imagery and detecting anomalies using a region-based CNN with ResNet-50 as the backbone. Although effective, reliance on region-based approaches limits the real-time capabilities of the system, especially when processing large-scale datasets or handling smaller objects such as bird droppings. Similarly, ref. [21] developed failure and fault analysis techniques using aerial imagery captured by UAVs, incorporating image processing techniques for detailed inspection of PV panels. While their approach effectively identifies hotspots caused by bird droppings using thermography, it lacks the capability to distinguish between hotspots resulting from partial shadowing or temporary faults. This limitation reduces its utility for cleaning operations, as the thermal camera treats bird-dropping hotspots as faults rather than identifying them specifically for targeted cleaning interventions. In another study, ref. [22] presented an encoder-decoder-based deep learning method for the accurate segmentation of bird droppings. Despite its effectiveness in pixel-level segmentation, the model’s dependency on computationally intensive architectures such as VGG-19 limits its applicability for real-time UAV-based operations.

YOLO series has gained popularity in UAV-based applications due to its single-stage architecture, which ensures high-speed inference and real-time processing. This is particularly advantageous in aerial imaging, where large batches need to be processed rapidly. The researchers used a multistage model using YOLOv3 and computer vision techniques to identify defects in solar panels from UAV-captured images [23]. Their approach used multispectral IR thermography and RGB imagery to detect anomalies such as soiling, bird droppings, and delamination in PV panels. While effective, the method’s reliance on multispectral imaging increases operational complexity and costs, making it less feasible for routine inspections. Similarly, ref. [24] presented a YOLO-based pipeline to effectively detect vegetation on PV panels to ensure efficient maintenance cycles. However, the study focuses primarily on vegetation and lacks a comprehensive approach to address other types of soil, such as dust and bird droppings, which are equally critical for PV efficiency. YOLO-inspired models are quite popular in other domains, including agricultural applications, such as monitoring the development of maize and weed crops [25,26,27]. In these studies, the researchers adapted YOLOv5 by introducing a bidirectional pyramid structure and additional detection heads, coupled with attention modules such as SimAM, to improve detection accuracy. Despite these advancements, these modifications are optimized for detecting small aerial objects, whereas soiling on PV panels typically involves larger patches such as dust and bird droppings. Ref. [25] proposed an automatic grapevine detection and counting pipeline using UAV-RGB imagery, which allows tracking of vineyard growth and development. Focus on single-class, small-object detection limits the approach’s effectiveness in addressing multiclass detection challenges with significant size differences, as seen in soiling-related anomalies. Furthermore, ref. [28] improved YOLOv5 for small object detection by integrating an additional detection head, Involution, and CBAM modules validated in the VisDrone19 dataset. However, the modifications in this study are tailored for datasets such as VisDrone, which consists of objects with distinctive spatial and color features. In contrast, soiling on PV panels involves multiclass detection challenges, with objects such as dust and bird droppings varying in size and lacking specific color or shape features. This disparity limits the effectiveness of the proposed approach for detecting soiling anomalies on top of PV panels.

In the context of small object detection, HP-YOLOv8, proposed by [29], enhances YOLOv8 with advanced feature fusion and loss functions to improve detection accuracy in remote sensing images. Although their model is tested on multiple datasets, including the VisDrone19 dataset [30], the requirement for soiling detection differs significantly. Soiling detection necessitates module-level localization within the aerial image rather than treating the entire image as a single unit. This distinction is critical to accurately identify soiling patterns and enable targeted cleaning strategies, highlighting the need for tailored solutions in this domain. In the latter part of 2024, YOLOv11 was introduced as the latest evolution in the YOLO family [15]. Based on the literature, the YOLO series has demonstrated varying strengths tailored to specific detection scenarios, making it one of the most versatile object detection architectures. YOLOv5, in particular, has been extensively studied and widely applied due to its robust performance for detecting larger objects. Its well-optimized architecture and adaptability to modifications have made it a popular choice for various applications, including the detection of soils on solar panels [28]. Moreover, its simplicity and computational efficiency make it ideal for tasks that require precise architectural customizations, such as the SDS-YOLO model proposed in this study.

In contrast, YOLOv8 and YOLOv11 reflect significant advancements in the YOLO series, particularly for small-object detection. YOLOv8 integrates enhanced feature extraction mechanisms and refined processing capabilities, enabling it to achieve high accuracy for objects with subtle spatial characteristics [31]. Meanwhile, YOLOv11 incorporates advanced attention modules and a lightweight architecture specifically designed to excel in detecting smaller and occluded objects. This design reduces computational overhead while improving the detection accuracy for challenging scenarios [32]. Unlike the objects in VisDrone19, the soiling dataset presents unique challenges for detection. PV panels lack distinct spatial and color features, and the two classes of soiling, dust and bird droppings, exhibit significant variations in patch sizes, insights from these YOLO variants were carefully evaluated. Ultimately, YOLOv5 was selected as the foundation for SDS-YOLO due to its adaptability and proven ability to support precise architectural customizations. Although existing studies have used general and aerial object detection techniques for defect or anomaly detection, few have addressed the unique challenges posed by multiclass soiling in solar photovoltaic panels. This study bridges this gap by presenting a specialized detection pipeline that leverages UAV imagery, state-of-the-art object detection models, and a highly customized architecture. This approach specifically addresses the challenges of detecting dust and bird droppings with high precision, recall, and good computational efficiency, ensuring relevance for PV maintenance and cleaning optimization. In this regard, the present study is related to the accurate localization of the solar panel soiling level to initiate optimal cleaning operations based on the detection results.

## 3. Proposed Methodology

The fundamental objective of this research is to accurately identify and localize the two main classes of soiling, dust and bird droppings, on solar panels using boundary boxes. The need for pixel-level granularity is mitigated by the standard cleaning operating procedure (SOP), which stipulates that the entire panel be cleaned if any soiling is detected. This operational requirement renders satellite-based image processing methods, such as those used by [23], less practical for our purposes. These methods often treat an entire solar installation as a single unit, which is not operationally feasible since cleaning all panels is inefficient when only a select few might be affected by soiling. An initial attempt to use a single end-to-end model based on YOLO for both PV panel and soiling detection did not yield satisfactory results. The dataset comprising rooftop PV installations in commercial warehouses and buildings includes various background objects, such as windows and sheds, that resemble PV structures. One of the key limitations of the YOLO series in this context is its inability to restrict detection to specific regions, such as the boundaries of PV panels. Without the capability to constrain the detection area, the model was frequently deceived by similar-looking structures in the background.

**Figure 1:**
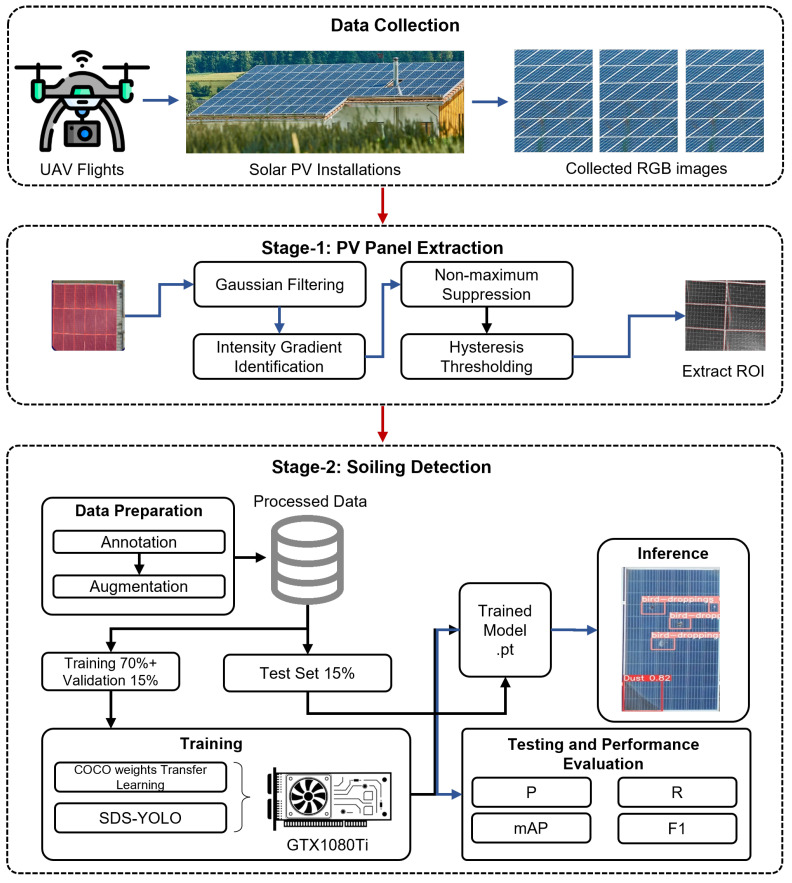
Block diagram of the proposed methodology for soiling detection.

The inability of YOLO to restrict detection within specific regions, such as the boundaries of PV panels, required a multistage approach. In this approach, PV panels are first detected separately, ensuring that subsequent soiling detection focuses solely on the panels. The methodology of this study is structured around a multistage architecture tailored for aerial imagery analysis. The first stage, the PV panel detector, is tasked with accurately detecting individual solar panels within an image. Panel-level detection is crucial for ensuring optimal cleaning efficiency, as the panel represents the smallest operational unit in a photovoltaic string. The second stage, the soiling detector, processes the images of the panels extracted from the first stage, focusing on detecting dust and bird droppings. This two-tier approach ensures precise localization of soiling, which is vital for targeted cleaning operations. The comprehensive process of the proposed model is illustrated in Figure 1.

**Figure 2:**
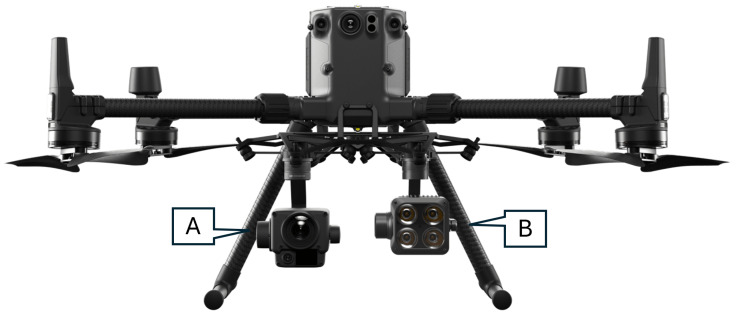
UAV platform for image acquisition: (**A**) Zenmuse H20; (**B**) Wingsland Z15 Gimbal Spotlight.

The unmanned aerial vehicle (UAV) platform used for image acquisition in this study was a DJI Matrice 300 RTK, as shown in Figure 2. This UAV was equipped with a suite of advanced instruments: a Zenmuse H20 and Z15 Gimbal Spotlight. Yellow Fin Robotics, Melbourne, Australia, has provided the necessary equipment for data collection. Although the Z15 Spotlight was part of the equipment setup to support low light conditions, most flights were conducted during the afternoon, when solar irradiation exceeded 800 W/m^2^, making additional lighting unnecessary. This allowed for extended flight time by conserving battery power. For the experiments, the UAV was operated within an altitude of 12–16 m, enabling the capture of high-resolution images. Details about the location of the experiments and the data acquisition specifics are concisely summarized in Table 1.

### 3.1. PV Panel Detection and Extraction of ROI (Stage 1)

The first stage focuses on accurately detecting PV panels within the complex imagery of PV installed on warehouse rooftops. Precise panel identification is essential if soiling is detected later. The Canny edge detector was specifically chosen because of its robustness in isolating object boundaries in complex scenes. In the context of our dataset, which comprises UAV-captured images of rooftop PV installations, background objects such as windows, sheds, and reflective surfaces often resemble solar panels. These objects can significantly interfere with the accuracy of soiling detection if they are not effectively isolated during preprocessing.

Canny edge detection excels in detecting well-defined continuous edges, which is particularly useful for identifying the rectangular boundaries of solar panels. This capability makes it more effective than edge detection techniques, such as Sobel or Prewitt, which may fail to preserve such clean, continuous boundaries in background noise. Moreover, its two-stage thresholding mechanism helps minimize false edges, ensuring that only the most relevant edges (i.e., the PV panel boundaries) are retained. This ensures accurate panel isolation from complex rooftop environments. The Canny edge detector working involves several key steps:

1. Smoothing: Initially, the input image undergoes smoothing using a Gaussian filter. This step reduces image noise and detail, preparing it for effective edge detection.
2. Gradient Calculation: The smoothed image is then processed using a Sobel operator to compute the image intensity gradient. The Sobel filter, applied in both horizontal and vertical directions, yields the first derivative of the image intensity, highlighting areas of potential edges.
3. Non-Maximum Suppression: This phase involves thinning the edges. Edges are points where the gradient magnitude is maximum, effectively suppressing all other gradient values (non-maximum) to zero.
4. Hysteresis Thresholding: The final step is to apply the hysteresis thresholding. This process differentiates between strong, true edges and weak, false edges, resulting in a more accurate edge map.

**Figure 3:**
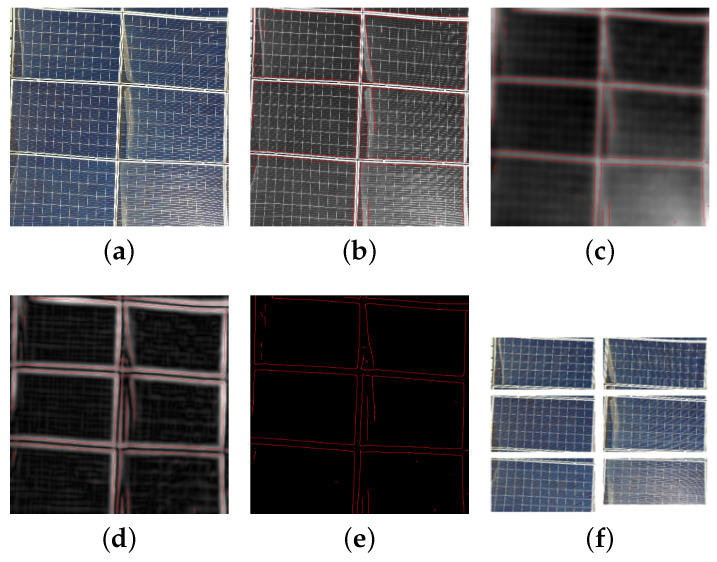
PV panel extraction process using Canny edge detector: (**a**) UAV-RGB captured image; (**b**) Smoothened image; (**c**) Gradient intensity applied; (**d**) Non-max suppression; (**e**) Hysteresis threshold; (**f**) Extracted PV Panel ROI.

Following these steps, the PV panels are distinctively outlined, and each panel is cropped from the original image to create individual Regions of Interest (ROIs). These ROIs are saved as separate entities and serve as inputs for the subsequent soiling detection stage. The detailed process of extracting PV panels via the detection of Canny edges is illustrated in Figure 3. This approach yielded high precision in isolating PV panels, which was validated during the data preparation phase. It also set the foundation for accurate soiling detection in the subsequent stages of the pipeline. The isolated panels were then used as input for the SDS-YOLO model, which performed the soiling detection task with significantly reduced background interference.

### 3.2. Soiling Detection (Stage 2)

#### 3.2.1. Data Preparation

The UAV image collection was meticulously performed to encapsulate the various conditions under which solar panels endure, including variations due to rain, windstorms, changes in irradiance, and differing fields of view. Given the importance of a high-quality dataset in a data-driven approach, our dataset was carefully curated from aerial images captured by UAVs of rooftop PV panels. These images were collected in various environmental conditions in the suburbs of Melbourne, providing a balanced representation of the two primary soil types: dust and bird droppings. A total of 300 aerial images. This is the first publicly available dataset designed specifically for multiclass soiling detection on top of PV panels, tailored for cleaning and maintenance optimization. Available: (https://www.ia-cobotics.com/research-projects/ai-driven-sola-panel-inspection, accessed on 10 January 2025).

To ensure high-quality imagery for accurate soiling detection, the UAV-based imaging process adhered to the following guidelines:

- Height: The images were captured at an optimal height of about 10 m, ensuring sufficient resolution to detect small soil patterns, such as bird droppings, while preserving a broader context for accurate localization.
- Orientation: Most of the images were captured in a nadir (downward facing) orientation to achieve uniform coverage of the photovoltaic panels. However, oblique angles were occasionally employed to better analyze the soil near the edges of the panel or the boundaries of the frame.
- Time of Day: Data collection was performed during the morning or late afternoon hours to minimize the impact of glare and reflections caused by harsh midday sunlight.
- Panel Conditions: The dataset comprises panels in varying conditions, including clean panels, dusty panels, and panels affected by bird droppings, ensuring a well-balanced dataset suitable for generalization.
- Imaging Settings: High-resolution settings were used for all images to ensure clarity and the ability to capture subtle soiling details. An overlap of 60–80% between consecutive images was maintained to provide complete coverage for large installations.

The subsequent step involved preparing these raw images for training the YOLOv5 model, necessitating a dataset comprising images with their corresponding bounding boxes and class labels. For the soiling detection component of our study, YOLOv5 was selected as the baseline object detection technique due to its modular design and ability to detect multiple classes simultaneously. This makes it well-suited for complex real-world tasks, such as soiling detection on photovoltaic (PV) panels.

#### 3.2.2. Data Annotation and Augmentation Process

YOLOv5 requires that each image be paired with a *.txt file containing the annotation box information. This research focuses on two classes of soiling, with the potential for multiple instances within a single image. The annotations have the following specifications:

- Format: Each row within a *.txt file represents a single object following the class format, x_center, y_center, width, and height.
- Normalization: The bounding box coordinates are normalized relative to the image dimensions.
- Zero-Indexed Classes: The classes are indexed from zero.

**Figure 4:**
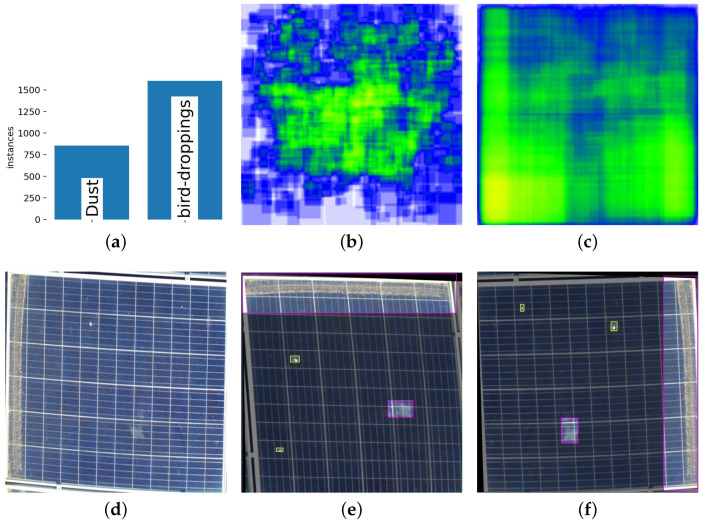
Visualization of labeled soiling dataset and augmented images: (**a**) Instance information of soiling dataset; (**b**) Heat map of bird-dropping instances; (**c**) Heatmap of dust instances; (**d**) PV Panel extracted before applying data augmentation; (**e**) Horizontally flipped, rotated 90°, and shear X = 5°, Y = 5° applied to (**d**); (**f**) Further rotated 90° and shear X = 4°, Y = 4° applied to (**e**).

We used the Roboflow labeling tool [33] to generate these specific ground truth labels. To optimize computational efficiency and maintain critical visual details, the images were resized to 640 × 640, according to the standard practice in aerial image processing. Additionally, all images were resized to a dimension of 640 × 640 pixels. Figure 4 illustrates some representative image samples from the soiling dataset along with the heat maps of the dust and bird-dropping classes.

To enhance the model’s generalization capabilities and improve its robustness against real-world variability, a comprehensive set of data augmentation techniques was employed. These techniques were carefully selected to address the unique challenges associated with UAV-captured images, which often vary in orientation, altitude, and environmental conditions. Geometric transformations, including vertical flips, 90-degree rotations, and horizontal and vertical shears of up to 10%, were applied to simulate various UAV flight paths and perspectives. Additionally, random translations and scaling adjustments were introduced to account for positional shifts and changes in apparent panel size caused by UAV altitude variations.

Photometric adjustments, such as variations in brightness, contrast, hue, and saturation, were applied to replicate lighting differences due to environmental factors such as shadows, overcast conditions, and direct sunlight. The addition of Gaussian noise was incorporated to simulate sensor imperfections and environmental disturbances, ensuring the robustness of the model against real-world artifacts. These augmentations not only increased the effective size of the dataset but also reduced the risk of overfitting, which is particularly important for the small size of the soiling dataset. Finally, the soiling dataset was reviewed multiple times to ensure the precision and consistency of the labels.

#### 3.2.3. YOLOv5 Model Configuration

The choice of YOLOv5 as the foundation for SDS-YOLO was directed by an extensive literature review and experimental evaluations. YOLOv5 is a well-established architecture known for its balance between speed and accuracy, making it ideal for modifications and optimizations, particularly in resource-constrained environments. Although YOLOv8 excels in detecting larger objects and YOLOv11 demonstrates improved capabilities for smaller object detection, our dataset includes a combination of small (bird droppings) and larger (dust) soiling features. This dual requirement motivated the selection of YOLOv5 as its flexibility and widespread adoption enable tailored enhancements like the integration of the Convolutional Block Attention Module (CBAM) and custom detection heads, which specifically address the unique challenges of multiclass soiling detection in PV panels.

The primary objective of this study is to achieve a higher detection rate in various soiling classes. However, a secondary but equally critical consideration is maintaining a good balance between model size and computational efficiency. In UAV-based applications, where large batches of data need to be processed efficiently, it is essential that the model has low GFLOPS to ensure reasonable inference times and energy-efficient operation. This requirement underscores the importance of tracking computational cost metrics like GFLOPS while designing the model, further justifying the adoption of YOLOv5 as a flexible and efficient baseline. The YOLOv5 model, central to our soiling detection methodology, is structured into three main components: the backbone, neck, and detection head. Among its variants for this study, we chose YOLOv5s as our baseline model due to its compact size, competent performance, and real-time inference capabilities, making it suitable for potential future deployment on edge devices like Jetson hardware. Recent research [34] has demonstrated YOLOv5s’ effectiveness in real-time multi-object detection and tracking, particularly within edge-accelerated systems, making it suitable for applications such as multi-drone operations.

**Figure 5:**
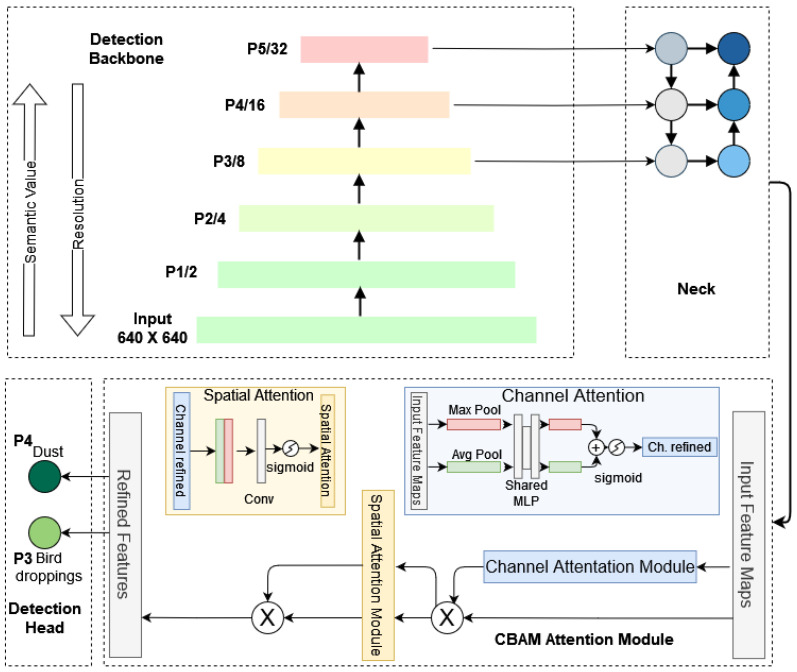
Model architecture of SDS-YOLO for soiling detection.

The YOLOv5 architecture, detailed in Figure 5, incorporates the CSPDarknet53 backbone for efficient feature extraction. It progressively downscales the input image, gathering contextual information at each level. The feature maps thus generated are then integrated by the neck, which employs skip connections to mitigate the vanishing-gradient problem. The detection head operates at three different scales to make predictions. For 640 × 640 images, feature maps at various scales are generated: P1 (320 × 320), P2 (160 × 160), P3 (80 × 80), P4 (40 × 40), and P5 (20 × 20). These feature maps serve as input for the YOLOv5 neck module. Convolutional operation of the backbone feature extraction can be represented as follows:

(1) $$Y\left(k,i,j\right)=\sum_{n=0}^{N-1}\sum_{m=0}^{M-1}W\left(n,m\right)\cdot X\left(i+n,j+m\right),$$

where *X* is the input feature map, *W* is the convolutional kernel, and *Y* is the output feature map. The *k* index refers to a specific output feature map with the filter’s N×M size, and *i* and *j* are the spatial coordinates.

The neck of YOLOv5 integrates the structures of the Feature Pyramid Network (FPN) and the Path Aggregation Network (PANet). FPN primarily comprises a bottom-up path, corresponding to the YOLOv5 backbone, and a top-down path. The Bottom-up path successively reduces the feature map size, enriching semantic information, while the Top-down path incrementally upsamples the smallest feature map, enhancing the semantic detail of lower-level features. Finally, feature maps with the same size on the two paths are laterally linked to increase semantic representation on multiple scales. The mathematical representation of these aggregation and upsampling processes is given in Equation (2).

(2) $$P_{i}=Conv\left(Upsample\left(P_{i+1}\right)+C_{i}\right).$$

where $P_{i}$ and $C_{i}$ represent the feature maps at different levels, and Conv represents a convolutional operation that refines the combined features. In the head, the upsampled feature maps are concatenated with features from the backbone to ensure seamless integration of spatial and semantic information. These feature maps undergo further processing through convolutional layers and CBAM modules, which emphasize the most critical features relevant to soiling detection.

YOLOv5 employs three prediction heads to target different object sizes: large (80 × 80), medium (40 × 40), and small (20 × 20) on a 640 × 640 resolution image. These heads operate on the respective feature maps, utilizing grids and anchors of varying aspect ratios to generate candidate bounding boxes. Subsequent application of Non-Maximum Suppression (NMS) refines these boxes, discarding overlaps and outputting the final detections, including box locations, sizes, and object confidence scores. The mathematical representation of the detection head is given by Equations (3) and (4).

(3) $$B=\sigma (Conv\left(P_{i}\right)).$$

where *B* represents the bounding box coordinates and class probabilities, σ is the sigmoid function, and Conv is a 1 × 1 convolution that outputs the bounding box attributes.

(4) $$O=\sigma \left(Conv\left(P_{i}\right)\right),\;C=\mathrm{softmax}\left(Conv\left(P_{i}\right)\right).$$

where *O* is the objectness score, *C* represents the class probabilities, and softmax normalizes the class prediction scores.

#### 3.2.4. CBAM Attention Mechanism

Attention mechanisms can dynamically highlight important features while ignoring irrelevant ones. This is particularly useful in multiclass detection, where different classes may have distinct features that need to be emphasized [35]. They can handle varying scales of objects within the same image, making them particularly effective for detecting small objects or objects at different scales. Attention mechanisms can enhance the accuracy of multiclass detection models by focusing on the most relevant parts of the input. Given the relatively small dataset size and the inherent variations across soiling classes, capturing subtle and critical feature information necessitated integrating a targeted attention mechanism. After extensive experimentation, it became evident that enhancing the model with a dedicated attention mechanism was crucial for improving detection rates. To address this, the Convolutional Block Attention Module (CBAM) was incorporated, enabling the model to focus selectively on essential spatial and channel-wise features relevant to soiling patterns. By recalibrating feature maps, CBAM highlights the most informative elements while mitigating challenges such as background noise, occlusions, and texture inconsistencies. This selective feature enhancement within the model’s intermediate layers significantly improves accuracy, enabling the model to better distinguish between dust and bird droppings, thereby enhancing overall detection performance. CBAM operates sequentially through Channel Attention and Spatial Attention mechanisms, each targeting feature recalibration from different perspectives.

Channel Attention Mechanism: The channel attention mechanism identifies the importance of each feature channel using global pooling operations (average and max pooling) to capture different perspectives of channel-wise significance. Given an input feature map $F\in R^{C\times H\times W}$ with *C* channels, height *H*, and width *W*, the channel attention module computes a channel attention map $M_{\mathrm{Channel}}\in R^{C\times 1\times 1}$ as follows:

(5) $$M_{c}\left(F\right)=\sigma ({\mathrm{Conv}}_{2}\left(\mathrm{ReLU}\left({\mathrm{Conv}}_{1}\left(\mathrm{AvgPool}\left(F\right)\right)\right)\right)+{\mathrm{Conv}}_{2}\left(\mathrm{ReLU}\left({\mathrm{Conv}}_{1}\left(\mathrm{MaxPool}\left(F\right)\right)\right)\right)$$

where σ denotes the sigmoid function and FC represents fully connected layers. The final output $F^{\prime }$ of the channel attention stage is obtained by applying $M_{\mathrm{Channel}}$ to *F*:

(6) $$F^{\prime }=M_{\mathrm{Channel}}\left(F\right)\cdot F$$

Spatial Attention Mechanism: The spatial attention mechanism applies attention across spatial dimensions to emphasize important spatial locations. Using $F^{\prime }$ from the channel attention stage, spatial attention produces an attention map $M_{\mathrm{Spatial}}\in R^{1\times H\times W}$ by performing average and max pooling along the channel axis and concatenating them:

(7) $$M_{\mathrm{Spatial}}\left(F^{\prime }\right)=\sigma \left(\mathrm{Conv}\left(\left[\mathrm{AvgPool}\left(F^{\prime }\right);\mathrm{MaxPool}\left(F^{\prime }\right)\right]\right)\right)$$

Here, Conv denotes a convolutional layer, σ is the sigmoid function, and [;] indicates channel-wise concatenation. The output $F_{\mathrm{CBAM}}$ after spatial attention is as follows:

(8) $$F_{\mathrm{CBAM}}=M_{\mathrm{Spatial}}\left(F^{\prime }\right)\cdot F^{\prime }$$

The overall CBAM operation for an input feature map *F* is thus the following:

(9) $$F_{\mathrm{CBAM}}=M_{\mathrm{Spatial}}\left(M_{\mathrm{Channel}}\left(F\right)\cdot F\right)\cdot \left(M_{\mathrm{Channel}}\left(F\right)\cdot F\right)$$

These equations are adapted from the original CBAM paper [36]. This lightweight and sequential attention module enables the network to focus on the most relevant channel and spatial features, thus improving feature representations for enhanced detection.

Strategic Placement of CBAM: CBAM modules were strategically placed before each detection head, allowing feature recalibration to occur immediately before the detection stage. This placement improves the relevance of the features that are fed to the detection heads, allowing each head to focus more effectively on the specific soiling type that it is responsible for identifying. The recalibrated feature maps, fine-tuned by channel and spatial attention, contain amplified, class-specific information essential for accurate classification and localization.

## 4. Experiments and Performance Analysis

This section briefly describes the implementation platform, experiments, and performance evaluation.

### 4.1. Implementation Platform

To ensure the reliability and reproducibility of the experimental results, all experiments were conducted using the same hardware and software configuration. The hardware setup consists of an Intel Core i7-9700F CPU (RMIT Robotics Lab, Melbourne, Australia) operating at 3.00 GHz, 32 GB of RAM, and 1.5 terabytes of Solid-State Disk. The graphics processing unit (GPU) utilized was an NVIDIA GTX1080Ti. The algorithm was implemented in Python 3.9 using the PyTorch framework version 2.1.0, and CUDA 12.1 was employed for accelerated model training. The proposed SDS-YOLO model is trained using a consistent set of hyperparameters throughout the training process. Precise information on these hyperparameters is provided in Section 4.3. In this study, the soiling dataset instances, shown in Figure 4a, clearly illustrate a class imbalance issue, where instances of bird droppings are more prevalent than dust. This class imbalance scenario makes the F1 score a more informative metric than precision, as it balances precision and recall. In general, recall, precision, mAP, and GFLOPS scores provide a more comprehensive performance evaluation and insight into the model’s performance. The details of these performance metrics are presented in Appendix A.

### 4.2. Loss, Anchor Size, and Training Parameters

YOLOv5 loss function comprises three components, objectness, bounding box, and class probability, which are expressed as follows:

(10) $$Loss=\alpha \times {\mathrm{Loss}}_{\mathrm{obj}}+\beta \times {\mathrm{Loss}}_{\mathrm{box}}+\gamma \times {\mathrm{Loss}}_{\mathrm{cls}.}$$

Cross Entropy Loss as a metric for evaluating the performance of classification predictions. In our study, we modified the classification loss function by using the weighted loss function to address the class imbalance by assigning different weights to the minority (dust) and majority (bird droppings) classes. Specifically, we assign higher weights to the minority class and lower weights to the majority class to ensure that the loss for the minority class is penalized more.

The modified loss function is given by the following:

(11) $$Loss_{\mathrm{cls}}=-\left[w_{1}\cdot y\cdot \log\left(p\right)+w_{2}\cdot \left(1-y\right)\cdot \log\left(1-p\right)\right],$$

where y=1,2 for class, *p* is the predicted probability, and $w_{1}$ and $w_{2}$ are the weights assigned to class 1 and class 2, respectively.

By adjusting the weights $w_{1}$ and $w_{2}$, we effectively penalize the loss for the minority class more heavily, thus addressing the imbalance and improving the model’s performance on the minority class. The weights $w_{1}$ and $w_{2}$ were determined empirically by iterative experimentation. Starting with an initial estimate based on the inverse frequency of the classes, we adjusted the weights by monitoring the model’s recall and precision metrics for both classes during training. After extensive experimentation, we found that the settings $w_{1}=0.3$ and $w_{2}=0.7$ yielded the best results in terms of detection accuracy. This approach ensured that the minority class contributed proportionally to the overall loss, improving its detection performance without significantly affecting the performance of the majority class. By penalizing the minority class loss more heavily, the model effectively addressed the class imbalance, resulting in improved detection performance for the dust class.

As mentioned in Section 3.2.2, the baseline YOLOv5s model employs three detection heads. However, due to the size discrepancy in our soiling dataset, the default detection head configuration performs sub-optimally. By assigning a detection head to each class, we ensured that each detection head was specialized and optimized for the unique features of its corresponding class. This simplifies the computational pipeline and reduces redundancy in feature extraction, making the architecture more efficient for the given dataset. Additionally, we modified the anchor sizes in SDS-YOLO instead of using the YOLOv5 auto-anchor feature. Custom anchor sizes for our soiling dataset are finalized through extensive testing. These optimal sizes, specifically tailored to our dataset, are listed in Table 2.

### 4.3. Dataset Preparation and Training Strategy

The dataset was randomly divided into training, testing, and validation sets in a 70%:15%:15% ratio, taking into account its relatively small size. We utilized pre-trained YOLOv5 weights from the COCO dataset to compensate for the limited training data. This transfer learning strategy allowed the model to leverage previously learned features, significantly reducing the time and data required for effective training. It also played a crucial role in enhancing the model’s ability to generalize from a relatively small dataset. For optimization, we used the Adam optimizer with an initial learning rate set to 0.01 and a final learning rate of 0.2, alongside a momentum of 0.937 and a weight decay of 0.0007 to prevent overfitting. The input images were resized to 640 × 640 pixels (3 channels), and the model was trained with a batch size of 16, which was selected to maximize GPU utilization while maintaining stable training. The training was conducted over 200 epochs. To further ensure time-efficient training, an early stopping strategy was implemented with a patience parameter set to 15 epochs, halting training if the model’s performance on the validation set did not improve for 15 consecutive epochs. The training configuration and hyperparameters are given in Table 3.

### 4.4. Overall Performance of SDS-YOLO Model

The experimental results in Table 4 demonstrate that our enhanced algorithm substantially improves over the YOLOv5 baseline to detect bird dropping and dust soiling on PV panels.

In particular, the recall metric for bird droppings has shown a significant improvement, increasing from 0.478 in the baseline model to 0.690 in the SDS-YOLO model. This boost in recall is crucial as it ensures that the majority of bird-dropping instances are detected, reducing the likelihood of soiling that can detract from the efficiency of solar panels. High recall is essential in applications like solar panel maintenance, where missed detections could lead to decreased energy output and higher cleaning costs.

In terms of precision, SDS-YOLO maintains strong performance, increasing bird droppings from 0.657 to 0.712, balancing improvement in recall. This results in an enhanced F1 score for bird droppings, increasing from 0.550 to 0.700, indicating a more robust and balanced detection capability that minimizes false negatives and false positives.

SDS-YOLO also exhibits improvements for the dust class, with recall increasing from 0.641 to 0.721 and precision increasing notably from 0.805 to 0.854. These gains lead to a higher F1 score for dust detection, improving from 0.710 in the baseline model to 0.781 in the SDS-YOLO model. The higher F1 score for dust suggests that the SDS-YOLO is particularly well suited to detect more challenging soiling patterns under various environmental conditions.

Beyond improvements in recall and precision, a comprehensive error analysis was performed to quantify the reductions in False Positives (FP) and False Negatives (FN) achieved by our SDS-YOLO at the beginning of the study, as detailed in Table 5. Taking into account differences in recall and precision, we calculate the reduction in FN as a direct result of improved recall with the formula

FN=(1−Recall)×TruePositives

Similarly, FP reduction was derived from enhanced precision, calculated as

FP=(1−Precision)×TruePositives

The error analysis demonstrates a substantial improvement in both FP and FN rates with SDS-YOLO compared to the baseline. Specifically, SDS-YOLO achieved a significant reduction in FN rate by approximately 30% for bird droppings and over 12% for dust, directly enhancing recall and ensuring fewer missed detections. Furthermore, the FP rate markedly improved, with reductions of around 12% for bird droppings and 15% for dust. These improvements indicate that our model captures more relevant soiling instances and reduces unnecessary detections, yielding a more balanced and accurate performance across both challenging soiling classes. For bird droppings, FNs arose primarily because of the small size and irregular shapes of the droppings, which occasionally caused them to blend with the background or be missed entirely during feature extraction. In contrast, FPs were observed when the textured regions of the panels, such as edge shading or residual streaks of cleaning, were misinterpreted as bird droppings. For the dust class, most errors were FPs, often caused by overlapping features or reflections that resembled dust patterns. One notable limitation leading to these errors is the model’s dependency on input resolution and lighting conditions, particularly in scenarios involving glare or uneven illumination. In addition, overlapping instances of bird droppings and dust on the same panel were occasionally misclassified as single soil types, reducing precision. Future work aims to mitigate these errors through several strategies: (1) Introducing higher-resolution inputs or adaptive resolution techniques during training to improve the detection of small objects. (2) Extending the dataset to include more instances of overlapping soiling types and variations in environmental conditions to improve the model’s robustness.

Modifications to the YOLOv5 architecture, including the targeted use of CBAM modules and customized anchor configurations, have significantly improved the detection accuracy in both soiling classes. These improvements are significant for applications where accurate detection of soiling types, such as bird droppings, significantly impacts panel efficiency and affects both short-term cleaning efficacy and long-term operational performance. The higher recall for both classes is especially advantageous for automated cleaning systems, which ensures a reliable detection rate that supports efficient maintenance scheduling.

### 4.5. Ablation Studies

Ablation studies were conducted to systematically assess the impact of each modification on the performance of the model, with a focus on improving the detection for the bird droppings class while maintaining robust performance for the dust class. These experiments evaluated the effect of adjusting the weights of the loss function, reducing detection heads, customizing anchor sizes, and integrating CBAM. The results are presented in Table 6, which shows the contribution of each modification to the effectiveness of the final model.

Starting with the baseline model, we recorded metrics using Binary Cross-Entropy (BCE) loss. The bird-droppings class showed a recall of 0.478, a precision of 0.657, an F1 score of 0.553, and a mAP50 of 0.498. For the dust class, the recall was 0.641, the precision was 0.805, the F1 score was 0.713, and mAP50 was 0.728. This model had a computational cost of 15.8 GFLOPS and 70.15 million parameters.

The first modification applied was class-weighted BCE loss, with weights set to [0.3, 0.7]. This adjustment led to an increase in recall for the bird-droppings class, reaching 0.586, while precision remained balanced at 0.585, yielding an F1 score of 0.585 and mAP50 of 0.552. Dust class performance saw a slight decrease in recall to 0.575 but retained a high precision of 0.829.

The second modification involved reducing the detection heads (RDH) from three to two, optimizing the model specifically for our soiling dataset. This change resulted in a bird droppings recall of 0.550, precision of 0.631, F1 score of 0.587, and mAP50 of 0.541, with dust class recall and precision of 0.583 and 0.807, respectively. This configuration achieved a notable reduction in computational cost to 14.3 GFLOPS and parameters down to 52.41 million, highlighting the model’s efficiency.

Further refinement was achieved by introducing custom anchor sizes (CA). This led to improved performance metrics, with the bird-droppings class achieving a recall of 0.664, a precision of 0.599, an F1 score of 0.630, and a mAP50 of 0.629. The dust class also showed improvements, with recall reaching 0.661, precision at 0.706, F1 score at 0.683, and mAP50 at 0.711. This configuration maintained an efficient 14.3 GFLOPS with 52.33 million parameters.

Although fully connected layers in the CBAM channel attention mechanism can be computationally demanding, our implementation ensures minimal overhead. To maintain a balance between accuracy and computational cost, we reduced the number of detection heads in the YOLOv5 architecture, significantly decreasing the size and complexity of the model. The inclusion of CBAM offers substantial performance improvements, particularly for the challenging bird-droppings class, despite adding a small computational cost. The final enhancement involved adding CBAM to recalibrate spatial and channel features within the detection head. This inclusion significantly elevated the performance of both classes, with the bird droppings class reaching a recall of 0.690, precision of 0.712, F1 score of 0.700, and mAP50 of 0.698. The dust class recall is also improved to 0.721, precision to 0.854, F1 score to 0.781, and mAP50 to 0.760. The size of the model saw a slight increase to 14.5 GFLOPS and 52.6 million parameters due to the addition of CBAM, reflecting a balanced improvement in detection accuracy and computational efficiency.

This stepwise approach in the ablation study reveals that each modification incrementally contributed to the overall performance of the model, and CBAM is particularly instrumental in refining the attention of features for soiling detection tasks.

### 4.6. Comparison with Other Models

To further validate the performance of the SDS-YOLO model, we performed a comparative analysis with other state-of-the-art models, including GBH-YOLOv5 from [24] and HIC-YOLOv5 from [28]. These studies were selected due to their modifications to the baseline YOLOv5, which improved detection performance, similar to our approach.

Specifically, ref. [24] adapted YOLOv5 by incorporating an additional detection head to improve the detection of aerial objects. The best-performing version of their study was reproduced using our soiling dataset, as their repository provided access to the necessary model configurations. Similarly, ref. [28] introduced modifications, including Ghost convolution and an additional detection head, to enhance YOLOv5’s performance for detecting small defects in solar PV modules using Electroluminescence images. This study is particularly relevant as it targets multiple small defect classes, analogous to the detection challenge posed by identifying bird droppings on solar panels. Table 7 presents the performance metrics of these reproduced models along with SDS-YOLO and the baseline.

From Table 7, it is evident that SDS-YOLO outperforms the baseline YOLOv5, YOLOv8, YOLOv11, and the reproduced GBH-YOLOv5 and HIC-YOLOv5 models in several metrics, particularly for the bird-dropping class. It should be noted that the methodologies presented in GBH-YOLOv5 and HIC-YOLOv5 exhibit exceptional performance within the context of their original application and dataset. However, our reimplementation of these methodologies in the new domain/application of soiling detection has yielded new results, highlighting the impact of contextual factors.

SDS-YOLO achieved the highest bird drop recall at 0.690, significantly exceeding baseline YOLOv5 (0.478), GBH-YOLOv5 (0.500), HIC-YOLOv5 (0.645), YOLOv8 (0.525) and YOLOv11 (0.570). The precision of bird droppings in SDS-YOLO reached 0.712, outperforming GBH-YOLOv5 (0.481), HIC-YOLOv5 (0.351), YOLOv8 (0.369), and YOLOv11 (0.439). Consequently, SDS-YOLO achieved the highest F1 score for bird droppings at 0.700, representing a substantial improvement over the other models. Furthermore, SDS-YOLO attained the highest mAP50 for bird droppings at 0.698, outperforming the baseline and all comparative models, including YOLOv8 and YOLOv11.

For the dust class, SDS-YOLO demonstrated robust performance, achieving a recall of 0.721, outperforming baseline YOLOv5 (0.641), YOLOv8 (0.655), YOLOv11 (0.650) and HIC-YOLOv5 (0.575). The precision of the dust class in SDS-YOLO was 0.854, exceeding the baseline (0.805), YOLOv8 (0.675), YOLOv11 (0.720), and HIC-YOLOv5 (0.655). SDS-YOLO’s F1 score for dust was 0.781, exceeding YOLOv8’s 0.766, YOLOv11’s 0.683, and close to the baseline YOLOv5’s 0.713. The dust mAP50 was 0.760, indicating competitive performance and showcasing SDS-YOLO’s adaptability across diverse soiling classes.

In terms of computational efficiency, SDS-YOLO demonstrates significant advantages, utilizing only 14.5 GFLOPS compared to baseline YOLOv5 (15.8 GFLOPS), GBH-YOLOv5 (16.5 GFLOPS), HIC-YOLOv5 (30.6 GFLOPS), YOLOv8 (28.6 GFLOPS) and YOLOv11 (21.3 GFLOPS). Although YOLOv11 boasts the lowest parameter count of 9.4 million, followed by YOLOv8’s 11.4 million, compared to SDS-YOLO’s 52.6 million, SDS-YOLO strikes a better balance between computational efficiency and detection performance, delivering significantly higher metrics for both classes. SDS-YOLO outperformed both YOLOv8 and YOLOv11 in terms of overall metrics, primarily due to its tailored modifications and attention mechanisms, which effectively address the diverse challenges of detecting multiple soiling classes. This comparative approach highlights the strengths and limitations of different YOLO versions while emphasizing the effectiveness of the proposed SDS-YOLO model for this specific application. This makes SDS-YOLO highly suitable for real-time resource-constrained and accurate detection is critical.

However, some limitations remain with our approach; certain regions may experience soiling patterns influenced by different climatic factors, such as sandstorms or industrial pollution, which are not represented in the current dataset. Additionally, the relatively small number of images limits the representation of rare or complex soiling scenarios, such as overlapping dust and bird droppings or soiling patterns on unconventional panel materials or colors. This limitation could affect the model’s generalizability when applied to other environments or panel types beyond those present in the dataset. Small dataset scenarios typically require a balance between precision (quality) and recall (quantity). In our case, recall was prioritized to ensure that the model captured the most relevant instances, even if this led to a minor drop in precision. For instance, while a higher recall minimizes the likelihood of missed detections, which are critical in detecting bird droppings or dust on solar panels, it often leads to a slight reduction in precision, translating into occasional over-detection. This trade-off is particularly justified in solar panel maintenance, where the cost of missing a soiling event (lower recall) is greater than the cost of unnecessary cleaning (lower precision).

A conservative approach (prioritizing recall) is preferred in real-world solar panel maintenance. Missing soiling events could lead to inefficiencies in power generation or potential long-term damage, whereas over-detection might only result in slightly higher cleaning costs. Thus, improved recall, particularly in the bird droppings class, aligns well with practical needs, ensuring effective and reliable detection.

Lastly, the performance differences between “bird droppings” and “dust” observed in other YOLO variants highlight that these models often perform well in one class while underperforming in the other. In contrast, SDS-YOLO demonstrates balanced performance in both soil types, achieving the lowest variance in recall and precision metrics between the two classes. This outcome underscores the intentional design and strength of SDS-YOLO’s custom modifications. This balanced performance ensures reliable detection in real-world applications where both small and large soil types significantly impact solar panel maintenance.

**Figure 6:**
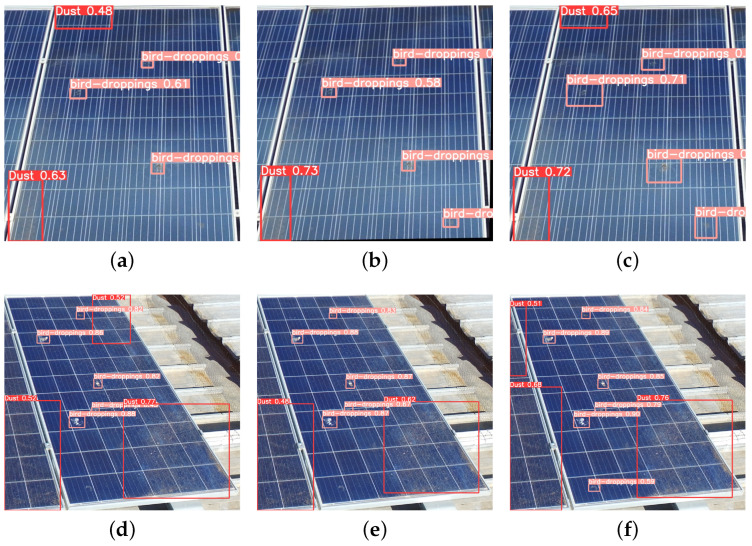
Example of multiple soiling class (dust and bird-dropping) detection results on the test subset: (**a**,**d**) YOLOv5; (**b**,**e**) HIC-YOLOv5; (**c**,**f**) Ours.

In general, SDS-YOLO demonstrates a superior detection performance and computational efficiency balance, underscoring its potential for practical, real-world applications in solar PV maintenance. Figure 6 visually illustrates the enhanced capability of our method in detecting more tiny bird droppings with better confidence compared to other approaches. This balance of detection performance and computational efficiency underscores the potential of SDS-YOLO for practical, real-world applications in solar PV maintenance.

## 5. Conclusions

This study presents a novel approach for addressing the challenge of soiling detection on solar PV panels, which uses aerial imagery captured by UAVs. Our approach employs a two-stage detection pipeline with a heavily customized network architecture based on YOLOv5, which is specifically designed to identify and localize two main soiling classes: dust and bird droppings. In the first stage, individual solar panels are precisely isolated from complex rooftop scenes, using the Canny edge detector, which ensures that subsequent soiling detection is focused solely on relevant areas. The second stage involves the SDS-YOLO model, which integrates targeted improvements, such as the CBAM and optimized anchor configurations, to enhance detection accuracy. This customized architecture demonstrated substantial gains, achieving a 40.2% improvement in mAP50 and a 26.6% increase in the F1 score for bird-dropping detection while maintaining competitive dust performance. These carefully calibrated modifications have strengthened the model’s ability to detect subtle soiling patterns, making it highly effective in capturing critical features and achieving balanced detection across both classes. The results underscore the potential of combining deep learning with UAV technology for automating the maintenance of solar PV panels. By enabling precise and timely soiling detection, our approach paves the way for optimized cleaning schedules, supporting the sustained operational efficiency of solar installations. Future work could focus on integrating this detection framework into semi-automated cleaning systems and adapting the model for real-time edge processing, further maximizing its impact and applicability in the renewable energy sector.

## Figures and Tables

**Table 1:** The specifications for the equipment used for aerial imagery collection.

Items	Expression
Location coordinates	−37.80, 144.96
Camera	Zenmuse H20
Maximum Resolution	5184 × 3888
Lens focal length	6.83–119.94 mm
Camera focus mode	MF/AF-C/AF-S with AE LOCK
UAV model	DJI Matrice 300 RTK
Flight range and endurance	7000 m, 55 min
Cruise speed	23 m/s
Height	6 m < h < 10 m
Solar PV Panel	Poly-crystalline
PV panel dimensions	1960 × 1308 mm
Max power	430 Watts

**Table 2:** Optimal Anchor box sizes for soiling detection.

Detection Heads	Anchor Frames
P3 (Bird droppings)	[15, 20], [42, 34], [119, 127]
P4 (Dust)	[238, 179], [89, 591], [295, 377]

**Table 3:** Hyperparameters used in training.

Hyperparameters	Value
Epochs	200
Input Size	640 × 640 (3 channel)
Batch Size	16
Learning Rate (initial, final)	(0.01, 0.2)
Momentum	0.937
Weight Decay	0.0007

**Table 4:** Performance metrics for YOLOv5 and SDS-YOLO models.

Configuration	Classes	Recall	Precision	F1	mAP
YOLOv5s	Bird droppings	0.478	0.657	0.55	0.498
	Dust	0.641	0.805	0.71	0.728
SDS-YOLO (ours)	Bird droppings	0.690	0.712	0.700	0.698
	Dust	0.721	0.854	0.781	0.760

**Table 5:** False Positive (FP) and False Negative (FN) reduction comparison between baseline (YOLOv5s) and SDS-YOLO models.

Classes	Baseline FN (%)	Baseline FP (%)	SDS-YOLO FN (%)	SDS-YOLO FP (%)	FN Improvement (%)	FP Improvement (%)
Bird droppings	52.2	34.3	31.0	28.8	40.6	16.1
Dust	35.9	19.5	27.9	14.6	22.3	25.1

**Table 6:** Ablation study results.

Model					Classes	Recall	Precision	F1	mAP50	GFLOPS	Params
YOLOv5s	BCE [0.3, 0.7]	RDH	CA	CBAM							
✓					Bird droppings	0.478	0.657	0.553	0.498	15.8	70.15 M
					Dust	0.641	0.805	0.713	0.728		
✓	✓				Bird droppings	0.586	0.585	0.585	0.552	15.8	70.15 M
					Dust	0.575	0.829	0.679	0.714		
✓		✓			Bird droppings	0.55	0.631	0.587	0.541	14.3	52.41 M
					Dust	0.583	0.807	0.676	0.656		
✓	✓	✓	✓		Bird droppings	0.664	0.599	0.63	0.629	14.3	52.33 M
					Dust	0.661	0.706	0.683	0.711		
✓	✓	✓	✓	✓	Bird droppings	0.690	0.712	0.700	0.698	14.5	52.6 M
					Dust	0.721	0.854	0.781	0.760		

**Table 7:** Comparison of performance metrics with other models.

Model	Classes	Recall	Precision	F1	mAP50	GFLOPS	Params
YOLOv5s	Bird droppings	0.478	0.657	0.553	0.498	15.8	70.15 M
	Dust	0.641	0.805	0.713	0.728		
YOLOv8	Bird droppings	0.525	0.369	0.433	0.412	28.6	11.14 M
	Dust	0.655	0.675	0.664	0.766		
YOLOv11	Bird droppings	0.57	0.439	0.495	0.437	21.3	9.4 M
	Dust	0.65	0.72	0.683	0.682		
GBH-YOLOv5 [24] (Reproduced)	Bird droppings	0.500	0.481	0.490	0.478	16.5	70.6 M
	Dust	0.650	0.811	0.721	0.705		
HIC-YOLOv5 [28] (Reproduced)	Bird droppings	0.645	0.351	0.454	0.498	30.6	92.9 M
	Dust	0.575	0.655	0.612	0.570		
SDS-YOLO (without CBAM) (ours)	Bird droppings	0.664	0.599	0.630	0.629	14.3	52.33 M
	Dust	0.661	0.706	0.683	0.711		
SDS-YOLO (ours)	Bird droppings	0.690	0.712	0.700	0.698	14.5	52.6 M
	Dust	0.721	0.854	0.781	0.760		

## Data Availability

Data are contained within the article.

## Appendix A. Performance Metrics

To assess the effectiveness of the SDS-YOLO algorithm for soiling detection, this study uses widely recognized standard metrics in the field: Precision (P), Recall (R), F1 score, and mean average precision (mAP). Below is an overview of definitions and formulations for each metric:

1. Precision

(A1) $$\mathrm{Precision}=\frac{TP}{TP+FP}$$

Precision measures the accuracy of positive predictions. It is the ratio of true positives (TP) to the sum of true positives and false positives (FP).

2. Recall

(A2) $$\mathrm{Recall}=\frac{TP}{TP+FN}$$

Recall measures the ability to find all relevant instances. It is the ratio of true positives (TP) to the sum of true positives and false negatives (FN).

3. F1 Score

(A3) $$F1=2\cdot \frac{\mathrm{Precision}\cdot \mathrm{Recall}}{\mathrm{Precision}+\mathrm{Recall}}$$

The F1 score is the harmonic mean of Precision and Recall, providing a single metric that balances both.

4. Average Precision (AP) and Mean Average Precision at IoU 0.50 (mAP50)

(A4) $$\mathrm{AP}=\int_{0}^{1}P\left(R\right)\;dR,\;\mathrm{mAP}50=\frac{1}{N}\sum_{i=1}^{N}AP_{i}$$

Average Precision (AP) summarizes the precision–recall curve as the area under the curve (AUC), calculated as the integral of precision (P) with respect to recall (R). The mean average precision at IoU 0.50 (mAP50) is the mean average precision at a 50% intersection above the Union threshold (IoU).
